# Potent neutralizing antibodies from COVID-19 patients define multiple targets of vulnerability

**DOI:** 10.1126/science.abc5902

**Published:** 2020-06-15

**Authors:** Philip J. M. Brouwer, Tom G. Caniels, Karlijn van der Straten, Jonne L. Snitselaar, Yoann Aldon, Sandhya Bangaru, Jonathan L. Torres, Nisreen M. A. Okba, Mathieu Claireaux, Gius Kerster, Arthur E. H. Bentlage, Marlies M. van Haaren, Denise Guerra, Judith A. Burger, Edith E. Schermer, Kirsten D. Verheul, Niels van der Velde, Alex van der Kooi, Jelle van Schooten, Mariëlle J. van Breemen, Tom P. L. Bijl, Kwinten Sliepen, Aafke Aartse, Ronald Derking, Ilja Bontjer, Neeltje A. Kootstra, W. Joost Wiersinga, Gestur Vidarsson, Bart L. Haagmans, Andrew B. Ward, Godelieve J. de Bree, Rogier W. Sanders, Marit J. van Gils

**Affiliations:** 1 Department of Medical Microbiology, Amsterdam UMC, University of Amsterdam, Amsterdam Institute for Infection and Immunity, 1105AZ Amsterdam, Netherlands.; 2 Department of Internal Medicine, Amsterdam UMC, University of Amsterdam, Amsterdam Institute for Infection and Immunity, 1105AZ Amsterdam, Netherlands.; 3 Department of Integrative Structural and Computational Biology, The Scripps Research Institute, La Jolla, CA 92037, USA.; 4 Department of Viroscience, Erasmus Medical Center, Rotterdam, 3015GD, Netherlands.; 5 Sanquin Research, Department of Experimental Immunohematology, Amsterdam, Netherlands and Landsteiner Laboratory, Amsterdam UMC, University of Amsterdam, 1006AD Amsterdam, Netherlands.; 6 IBIS Technologies BV, 7521PR Enschede, Netherlands.; 7 Department of Virology, Biomedical Primate Research Centre, 2288GJ Rijswijk, Netherlands.; 8 Department of Experimental Immunology, Amsterdam UMC, University of Amsterdam, Amsterdam Institute for Infection and Immunity, 1105AZ Amsterdam, Netherlands.; 9 Department of Microbiology and Immunology, Weill Medical College of Cornell University, New York, NY 10021, USA.

## Abstract

The rapid spread of severe acute respiratory syndrome coronavirus 2 (SARS-CoV-2) has had a large impact on global health, travel, and economy. Therefore, preventative and therapeutic measures are urgently needed. Here, we isolated monoclonal antibodies from three convalescent coronavirus disease 2019 (COVID-19) patients using a SARS-CoV-2 stabilized prefusion spike protein. These antibodies had low levels of somatic hypermutation and showed a strong enrichment in VH1-69, VH3-30-3, and VH1-24 gene usage. A subset of the antibodies was able to potently inhibit authentic SARS-CoV-2 infection at a concentration as low as 0.007 micrograms per milliliter. Competition and electron microscopy studies illustrate that the SARS-CoV-2 spike protein contains multiple distinct antigenic sites, including several receptor-binding domain (RBD) epitopes as well as non-RBD epitopes. In addition to providing guidance for vaccine design, the antibodies described here are promising candidates for COVID-19 treatment and prevention.

## Sites of vulnerability in SARS-CoV-2

Antibodies that neutralize severe acute respiratory syndrome coronavirus 2 (SARS-CoV-2) could be an important tool in treating coronavirus disease 2019 (COVID-19). Brouwer *et al.* isolated 403 monoclonal antibodies from three convalescent COVID-19 patients. They show that the patients had strong immune responses against the viral spike protein, a complex that binds to receptors on the host cell. A subset of antibodies was able to neutralize the virus. Competition and electron microscopy studies showed that these antibodies target diverse epitopes on the spike, with the two most potent targeting the domain that binds the host receptor.

*Science*, this issue p. 643

## Teaser

Isolation of 403 monoclonal antibodies from COVID-19 patients revealed convergent gene usage and multiple target epitopes.

The rapid emergence of three novel pathogenic human coronaviruses in the past two decades has caused major concerns. The latest, severe acute respiratory syndrome coronavirus 2 (SARS-CoV-2), is responsible for >3 million infections and 230,000 deaths worldwide as of 1 May 2020 (*1*). Coronavirus disease 2019 (COVID-19), caused by SARS-CoV-2, is characterized by mild, flu-like symptoms in most patients. However, severe cases can present with bilateral pneumonia that may rapidly deteriorate into acute respiratory distress syndrome (*2*). With high transmission rates and no proven curative treatment available, health care systems are severely overwhelmed, and stringent public health measures are in place to prevent infection. Safe and effective treatment and prevention measures for COVID-19 are urgently needed.

During the outbreak of the first severe acute respiratory syndrome coronavirus (SARS-CoV) and Middle Eastern respiratory syndrome coronavirus (MERS-CoV), plasma of recovered patients containing neutralizing antibodies (NAbs) was used as a safe and effective treatment option to decrease viral load and to reduce mortality in severe cases (*3*, *4*). Recently, a small number of COVID-19 patients treated with convalescent plasma showed clinical improvement and a decrease in viral load (*5*). An alternative treatment strategy would be to administer purified monoclonal antibodies (mAbs) with neutralizing capacity. mAbs can be thoroughly characterized in vitro and expressed in large quantities. In addition, because of the ability to control dosing and composition, mAb therapy has improved efficacy over convalescent plasma treatment and prevents the potential risks of antibody-dependent enhancement (ADE) from non-neutralizing or poorly neutralizing Abs present in plasma that consists of a polyclonal mixture (*6*). Recent studies with patients infected with the Ebola virus highlight the superiority of mAb treatment over convalescent plasma treatment (*7*, *8*). Moreover, mAb therapy has been proven safe and effective against influenza virus, rabies virus, and respiratory syncytial virus (RSV) (*9*–*11*).

The main target for NAbs on coronaviruses is the spike (S) protein, a homotrimeric glycoprotein that is anchored in the viral membrane. Recent studies have shown that the S protein of SARS-CoV-2 bears considerable structural homology to that of SARS-CoV, consisting of two subdomains: the N-terminal S1 domain, which contains the N-terminal domain (NTD) and the RBD for the host cell receptor angiotensin-converting enzyme-2 (ACE2), and the S2 domain, which contains the fusion peptide (*12*, *13*). Similar to other viruses containing class 1 fusion proteins (e.g., HIV-1, RSV, and Lassa virus), the S protein undergoes a conformational change and proteolytic cleavage upon host cell receptor binding from a prefusion to a postfusion state, enabling merging of viral and target cell membranes (*14*, *15*). When expressed as recombinant soluble proteins, class 1 fusion proteins generally have the propensity to switch to a postfusion state. However, most NAb epitopes present in the prefusion conformation (*16*–*18*). The recent successes of isolating potent NAbs against HIV-1 and RSV using stabilized prefusion glycoproteins reflect the importance of using the prefusion conformation for isolating and mapping mAbs against SARS-CoV-2 (*19*, *20*).

Early efforts at obtaining NAbs focused on reevaluating SARS-CoV–specific mAbs isolated after the 2003 outbreak that might cross-neutralize SARS-CoV-2 (*21*, *22*). Although two mAbs were described to cross-neutralize SARS-CoV-2, most SARS-CoV NAbs did not bind SARS-CoV-2 S protein or neutralize SARS-CoV-2 virus (*12*, *21*–*23*). More recently, the focus has shifted from cross-neutralizing SARS-CoV NAbs to the isolation of new SARS-CoV-2 NAbs from recovered COVID-19 patients (*24*–*28*). S protein fragments containing the RBD have yielded multiple NAbs that can neutralize SARS-CoV-2 by targeting different RBD epitopes (*24*–*28*). In light of the rapid emergence of escape mutants in the RBD of SARS-CoV and MERS, monoclonal NAbs targeting epitopes other than the RBD are a valuable component of any therapeutic antibody cocktail (*29*, *30*). Indeed, therapeutic antibody cocktails with a variety of specificities have been used successfully against Ebola virus disease (*7*) and are being tested widely in clinical trials for HIV-1 (*31*). NAbs targeting non-RBD epitopes have been identified for SARS-CoV and MERS, supporting the rationale for sorting mAbs using the entire ectodomain of the SARS-CoV-2 S protein (*32*). In addition, considering the high sequence identity between the S2 subdomains of SARS-CoV-2 and SARS-CoV, using the complete S protein ectodomain instead of only the RBD may allow the isolation of mAbs that cross-neutralize different β-coronaviruses (*33*). In an attempt to obtain mAbs that target both RBD and non-RBD epitopes, we set out to isolate mAbs using the complete prefusion S protein ectodomain of SARS-CoV-2.

## Phenotyping SARS-CoV-2–specific B cell subsets

**Fig. 1:**
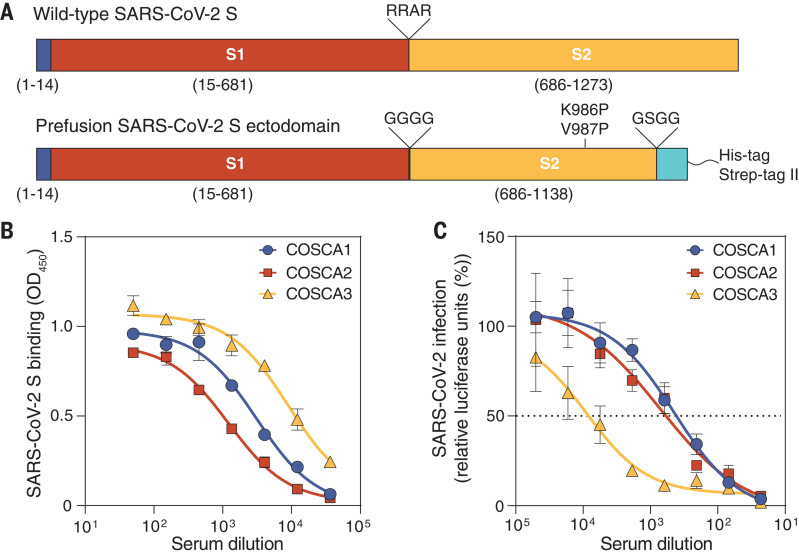
Design of SARS-CoV-2 S protein and serology of COSCA1, COSCA2, and COSCA3. (**A**) (Top) Schematic overview of the authentic SARS-CoV-2 S protein with the signal peptide shown in blue and the S1 (red) and S2 (yellow) domains separated by a furin-cleavage site (RRAR; top). (Bottom) Schematic overview of the stabilized prefusion SARS-CoV-2 S ectodomain, where the furin cleavage site is replaced with a glycine linker (GGGG), two proline mutations are introduced (K986P and V987P), and a trimerization domain (cyan) preceded by a linker (GSGG) is attached. (**B**) Binding of sera from COSCA1, COSCA2, and COSCA3 to prefusion SARS-CoV-2 S protein as determined by ELISA. The mean values and SDs of two technical replicates are shown. (**C**) Neutralization of SARS-CoV-2 pseudovirus by heat-inactivated sera from COSCA1, COSCA2, and COSCA3. The mean and SEM of at least three technical replicates are shown. The dotted line indicates 50% neutralization.

We collected a single blood sample from three polymerase chain reaction–confirmed SARS-CoV-2–infected individuals (COSCA1, COSCA2, and COSCA3) ~4 weeks after symptom onset. COSCA1 (a 47-year-old male) and COSCA2 (a 44-year-old female) showed symptoms of an upper respiratory tract infection and mild pneumonia, respectively (Table 1). Both remained in home isolation during the course of COVID-19 symptoms. COSCA3, a 69-year-old male, developed a severe pneumonia and became respiratory insufficient 1.5 weeks after symptom onset, requiring admission to the intensive care unit for mechanical ventilation. To identify S protein–specific antibodies in the sera obtained from all three patients, we generated soluble, prefusion-stabilized S proteins of SARS-CoV-2 using stabilization strategies previously described for S proteins of SARS-CoV-2 and other β-coronaviruses (Fig. 1A) (*12*, *34*). As demonstrated by the size-exclusion chromatography trace, SDS–polyacrylamide gel electrophoresis (PAGE), and blue native PAGE, the resulting trimeric SARS-CoV-2 S proteins were of high purity (fig. S1, A and B). Sera from all patients showed strong binding to the S protein of SARS-CoV-2 in an enzyme-linked immunosorbent assay **(**ELISA), with end-point titers of 13,637, 6133, and 48,120 for COSCA1, COSCA2, and COSCA3, respectively (Fig. 1B), and showed cross-reactivity to the S protein of SARS-CoV (fig. S1C). COSCA1, COSCA2, and COSCA3 had varying neutralizing potencies against SARS-CoV-2 pseudovirus, with 50% inhibition of virus infection (ID_50_) values of 383, 626, and 7645, respectively (Fig. 1C), and similar activities against authentic virus (fig. S1D). In addition, all sera showed cross-neutralization of SARS-CoV pseudovirus and authentic SARS-CoV virus, albeit with low potency (fig. S1, E and F). The potent S protein–binding and -neutralizing responses observed for COSCA3 are consistent with earlier findings showing that severe disease is associated with a strong humoral response (*35*). On the basis of these strong serum binding and neutralization titers, we sorted SARS-CoV-2 S protein–specific B cells for mAb isolation from COSCA1, COSCA2, and COSCA3.

**Table 1:** Patient characteristics, symptoms of COVID-19, treatment modalities, and sampling time points of three SARS-CoV-2–infected patients.

		**COSCA1**	**COSCA2**	**COSCA3**
**Patient characteristics**				
Age (years)		47	44	69
Gender		Male	Female	Male
Comorbidities		None	None	None
**Symptoms, from onset to relief, days**				
Fever (>38°C)		4–10	1–4	6–18
Coughing		2–35	3–17	1–20
Sputum production		2–35	No	1–20
Dyspnea		4–24	No	No
Sore throat		1–5	5–17	No
Rhinorrhea		2–34	5–17	No
Anosmia		No	5–17	No
Myalgia		No	1–4	6–18
Headache		No	No	1–18
Other		No	No	Delirium
**Treatment modalities, treatment period, days**				
Hospital admission		No	No	8–24
ICU admission		No	No	11–18
Oxygen therapy		No	No	8–24
	Intubation	No	No	11–16
Dialysis		No	No	No
Drug therapy				
	Antiviral	No	No	No
	Antibiotic	No	No	Cefotaxime, 8–12 Ciprofloxacin, 8–11
	Immunomodulatory	No	No	No
	NSAIDs	No	No	No
**Sampling time point, days after symptom onset**		27	28	23

**Fig. 2:**
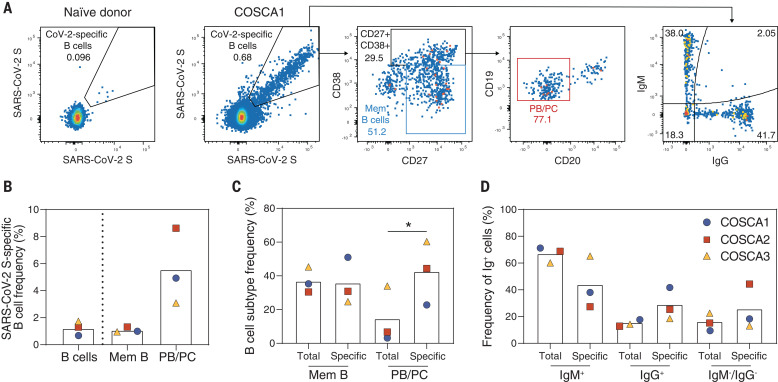
Characterization of SARS-CoV-2 S protein–specific B cells derived from COSCA1, COSCA2, and COSCA3. (**A**) Representative gates of SARS-CoV-2 S protein–specific B cells shown for a naïve donor (left panel) or COSCA1 (middle left panel). Each dot represents a B cell. The gating strategy to identify B cells is shown in fig. S2. From the total pool of SARS-CoV-2 S protein–specific B cells, CD27^+^CD38^–^ memory B cells (Mem B cells; blue gate) and CD27^+^CD38^+^ B cells were identified (middle panel). From the latter gate, PBs/PCs (CD20^–^; red gate) could be identified (middle right panel). SARS-CoV-2 S protein–specific B cells were also analyzed for their IgG or IgM isotype (right panel). (**B**) Frequency of SARS-CoV-2 S protein–specific B cells in total B cells, Mem B cells, and PBs/PCs. Symbols represent individual patients, as shown in (D). (**C**) Comparison of the frequency of Mem B cells (CD27^+^CD38^–^) and PB/PC cells (CD27^+^CD38^+^CD20^–^) between the specific (SARS-CoV2 S^++^) and nonspecific B cells (gating strategy is shown in fig. S2). Symbols represent individual patients, as shown in (D). Statistical differences between two groups were determined using paired *t* test (*P = 0.034). (**D**) Comparison of the frequency of IgM^+^, IgG^+^, and IgM^–^IgG^–^ B cells in specific and nonspecific compartments. Bars represent means; symbols represent individual patients.

Peripheral blood mononuclear cells were stained dually with fluorescently labeled prefusion SARS-CoV-2 S proteins and analyzed for the frequency and phenotype of specific B cells by flow cytometry (Fig. 2A and fig S2). The analysis revealed a frequency ranging from 0.68 to 1.74% of S protein–specific B cells (S-AF647^+^, S-BV421^+^) among the total pool of B cells (CD19^+^Via-CD3^–^CD14^–^CD16^–^), (Fig. 2B). These SARS-CoV-2 S protein–specific B cells showed a predominant memory (CD20^+^CD27^+^) and plasmablasts/plasma cells (PBs/PCs) (CD20^–^CD27^+^CD38^+^) phenotype. We observed a threefold higher percentage of PBs/PCs for SARS-CoV-2 S protein–specific B cells compared with total B cells (*P* = 0.034), indicating an enrichment of specific B cells in this subpopulation (Fig. 2C). COSCA3, who experienced severe symptoms, showed the highest frequency of PBs/PCs in both total (34%) and specific (60%) B cells (Fig. 2C and fig. S2). As expected, the SARS-CoV-2 S protein–specific B cells were enriched in the immunoglobulin G–positive (IgG^+^) and IgM^–^/IgG^–^ (most likely representing IgA^+^) B cell populations, although a substantial portion of the specific B cells were IgM^+^, particularly for COSCA3 (Fig. 2D).

## Genotypic signatures of the SARS-CoV-2–specific antibody response

**Fig. 3:**
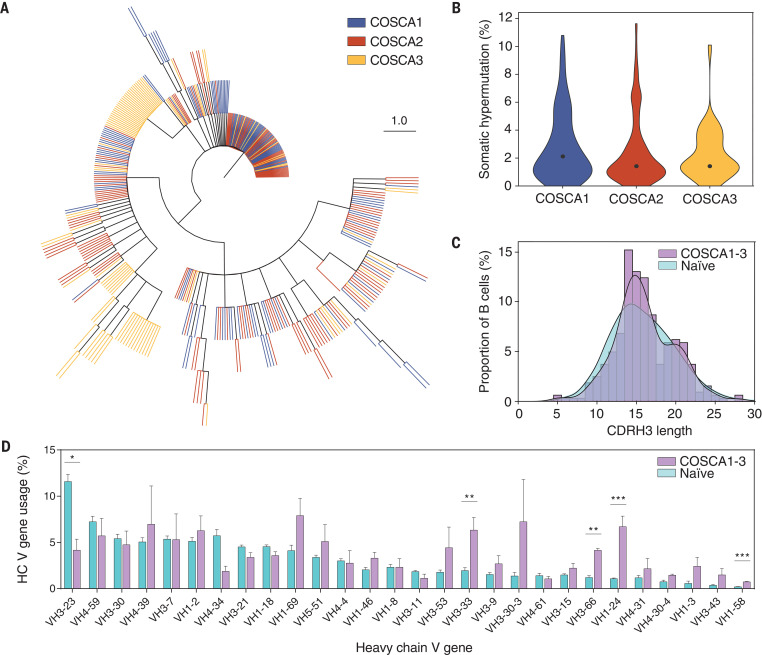
Genotypic characterization of SARS-CoV-2 S protein–specific B cell receptors. (**A**) Maximum-likelihood phylogenetic tree of 409 isolated paired B cell receptor HCs. Each color represents sequences isolated from different patients (COSCA1, COSCA2, and COSCA3). (**B**) Violin plot showing SHM levels (%; nucleotides) per patient. The dot represents the median SHM percentage. (**C**) Distribution of CDRH3 lengths in B cells from COSCA1, COSCA2, and COSCA3 (purple, *n* = 323) versus a representative naïve population from three donors (cyan, *n* = 9.791.115) (*37*). (**D**) Bar graphs showing the mean (± SEM) VH gene usage (%) in COSCA1, COSCA2, and COSCA3 (purple, *n* = 323) versus a representative naïve population (cyan, *n* = 363,506,788). The error bars represent the variation between different patients (COSCA1, COSCA2, and COSCA3) or naïve donors (*37*). Statistical differences between two groups were determined using unpaired *t* tests (with Holm–Sidak correction for multiple comparisons, adjusted *P* values: *P < 0.05; **P < 0.01; ***P < 0.001).

SARS-CoV-2 S protein–specific B cells were subsequently single-cell sorted for sequencing and mAb isolation. In total, 409 heavy chain (HC) and light chain (LC) pairs were obtained from the sorted B cells of the three patients (137, 165, and 107 from COSCA1, COSCA2, and COSCA3, respectively), of which 323 were unique clonotypes. Clonal expansion occurred in all three patients (Fig. 3A) but was strongest in COSCA3, where it was dominated by HC variable (VH) regions VH3-7 and VH4-39 (34 and 32% of SARS-CoV-2 S protein–specific sequences, respectively). Even though substantial clonal expansion occurred in COSCA3, the median somatic hypermutation (SHM) was 1.4%, with similar SHM in COSCA1 and COSCA2 (2.1 and 1.4%, respectively) (Fig. 3B). These SHM levels are similar to those observed in response to infection with other respiratory viruses (*36*).

A hallmark of antibody diversity is the heavy chain complementarity-determining region 3 (CDRH3). Because the CDRH3 is composed of V, D, and J gene segments, it is the most variable region of an antibody in terms of both amino acid composition and length. The average length of CDRH3 in the naïve human repertoire is 15 amino acids (*37*), but for a subset of influenza virus and HIV-1 broadly neutralizing antibodies, long CDRH3 regions of 20 to 35 amino acids are crucial for high-affinity antigen–antibody interactions (*38*, *39*). Even though the mean CDRH3 length of isolated SARS-CoV-2 S protein–specific B cells did not differ substantially from that of a naïve population (*37*), we observed a significant difference in the distribution of CDRH3 length (two-sample Kolmogorov–Smirnov test, *P* = 0.006) (Fig. 3C). This difference in CDRH3 distribution can largely be attributed to an enrichment of longer (~20 amino acid) CDRH3s, leading to a bimodal distribution as opposed to the bell-shaped distribution that was observed in the naïve repertoire (Fig. 3C and fig. S3).

Next, to determine SARS-CoV-2–specific signatures in B cell receptor repertoire usage, we compared ImmunoGenetics (IMGT) database–assigned unique germline V regions from the sorted SARS-CoV-2 S protein–specific B cells with the well-defined extensive germline repertoire in the naïve population (Fig. 3D) (*37*). Multiple VH genes were enriched in COSCA1, COSCA2, and COSCA3 compared with the naïve repertoire, including VH3-33 (*P* = 0.009) and VH1-24 (*P* < 0.001) (Fig. 3D). Even though the enrichment of VH1-69 was not significant (*P* > 0.05), it should be noted that an enrichment of VH1-69 has been shown in response to a number of other viral infections, including influenza virus, hepatitis C virus, and rotavirus (*40*), and an enrichment of VH3-33 was observed in response to malaria vaccination, whereas the enrichment of VH1-24 appears to be specific for COVID-19 (Fig. 3D) (*41*). By contrast, VH4-34 (*P* > 0.05) and VH3-23 (*P* = 0.018) were substantially underrepresented in SARS-CoV-2–specific sequences compared with the naïve population. Although usage of most VH genes was consistent between COVID-19 patients, VH3-30-3 and VH4-39 in particular showed considerable variability. Thus, upon SARS-CoV-2 infection, the S protein recruits a subset of B cells from the naïve repertoire enriched in specific VH segments and CDRH3 domains.

## Identification of unusually potent SARS-CoV-2–neutralizing antibodies

**Fig. 4:**
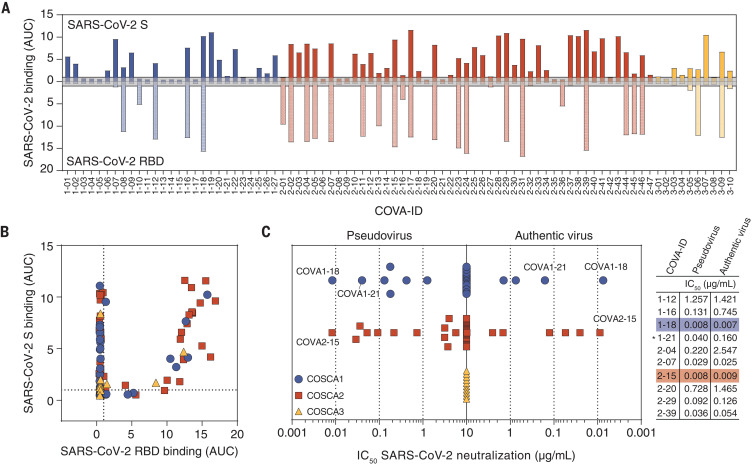
Phenotypic characterization of SARS-CoV-2 S protein–specific mAbs. (**A**) Bar graph depicting the binding of mAbs from COSCA1 (blue), COSCA2 (red), and COSCA3 (yellow) to SARS-CoV-2 S protein (dark shading) and SARS-CoV-2 RBD (light shading) as determined by ELISA. Each bar indicates the representative area under the curve (AUC) of the mAb indicated below from two experiments. The gray area represents the cutoff for binding (AUC = 1). The maximum concentration of mAb tested was 10 μg/ml. (**B**) Scatter plot depicting the binding of mAbs from COSCA1, COSCA2, and COSCA3 [see (C) for color coding] to SARS-CoV-2 S protein and SARS-CoV-2 RBD as determined by ELISA. Each dot indicates the representative AUC of a mAb from two experiments. (**C**) Midpoint neutralization concentrations (IC_50_) of SARS-CoV-2 pseudovirus (left) or authentic SARS-CoV-2 virus (right). Each symbol represents the IC_50_ of a single mAb. For comparability, the highest concentration was set to 10 μg/ml, although the actual start concentration for the authentic virus neutralization assay was 20 μg/ml. The IC_50_s for pseudotyped and authentic SARS-CoV-2 virus of a selection of potently neutralizing RBD and non-RBD–specific mAbs (with asterisk) are shown in the adjacent table. Colored shading indicates the most potent mAbs from COSCA1, COSCA2, and COSCA3.

Subsequently, all HC and LC pairs were transiently expressed in human embryonic kidney (HEK) 293T cells and screened for binding to SARS-CoV-2 S protein by ELISA. A total of 84 mAbs that showed high-affinity binding were selected for small-scale expression in HEK 293F cells and purified (table S1). We obtained few S protein–reactive mAbs from COSCA3, possibly because most B cells from this individual were IgM^+^, whereas cloning into an IgG backbone nullified avidity contributions to binding and neutralization present in the serum. To gain insight in the immunodominance of the RBD as well as its ability to cross-react with SARS-CoV, we assessed the binding capacity of these mAbs to the prefusion S proteins and the RBDs of SARS-CoV-2 and SARS-CoV using ELISA. Of the 84 mAbs tested, 32 (38%) bound to the SARS-CoV-2 RBD (Fig. 4, A and B), with seven mAbs (22%) showing cross-binding to SARS-CoV RBD (fig. S4A). We also observed 33 mAbs (39%) that bound strongly to SARS-CoV-2 S but did not bind the RBD, of which 10 mAbs (30%) also bound to the S protein of SARS-CoV (Fig. 4, A and B). Notably, some mAbs that bound very weakly to soluble SARS-CoV-2 S protein in ELISA showed strong binding to membrane-bound S protein, implying that their epitopes are presented poorly on the stabilized soluble S protein or that avidity is important for their binding (table S1). Surface plasmon resonance (SPR) assays confirmed binding of 77 mAbs to S protein and 21 mAbs to the RBD with binding affinities in the nanomolar to picomolar range (table S1).

All 84 mAbs were subsequently tested for their ability to block infection. A total of 19 mAbs (23%) inhibited SARS-CoV-2 pseudovirus infection with varying potencies (Fig. 4C) and, of these, 14 (74%) bound the RBD. Seven of the 19 mAbs could be categorized as potent neutralizers [median inhibitory concentration (IC_50_) < 0.1 μg/ml], six as moderate neutralizers (IC_50_ = 0.1 to 1 μg/mL), and six as weak neutralizers (IC_50_ = 1 to 10 μg/ml). With an IC_50_ of 0.008 μg/ml, the RBD-targeting antibodies COVA1-18 and COVA2-15 in particular were unusually potent. However, they were quite different in other aspects, such as their HC V gene usage (VH3-66 versus VH3-23), LC usage (VL7-46 versus VK2-30), HC sequence identity (77%), and CDRH3 length (12 versus 22 amino acids). Seventeen of the mAbs also interacted with the SARS-CoV S and RBD proteins and two of these cross-neutralized the SARS-CoV pseudovirus (IC_50_ = 2.5 μg/ml for COVA1-16 and 0.61 μg/ml for COVA2-02; fig. S4B), with COVA2-02 being more potent against SARS-CoV than against SARS-CoV-2. Next, we assessed the ability of the 19 mAbs to block infection of authentic SARS-CoV-2 virus (Fig. 4C and fig. S4C). Although previous reports suggested a decrease in neutralization sensitivity of primary SARS-CoV-2 compared with pseudovirus (*25*, *27*, *28*), we observed very similar potencies for seven of the 19 NAbs, including the most potent NAbs (IC_50_ = 0.007 and 0.009 μg/ml for COVA1-18 and COVA2-15, respectively; Fig. 4C). NAbs COVA1-18, COVA2-04, COVA2-07, COVA2-15, and COVA2-39 also showed strong competition with ACE2 binding, illustrating that blocking ACE2 binding is their likely mechanism of neutralization (fig. S4D). The RBD-targeting mAb COVA2-17, however, showed incomplete competition with ACE2. This corroborates previous observations that the RBD encompasses multiple distinct antigenic sites, some of which do not involve blocking of ACE2 binding (*23*, *25*, *26*). The non-RBD NAbs all bear substantially longer CDRH3s compared with RBD NAbs (fig. S4E), suggesting a convergent, CDRH3-dependent contact between antibody and epitope.

## Multiple targets of vulnerability on the SARS-CoV-2 S protein

**Fig. 5:**
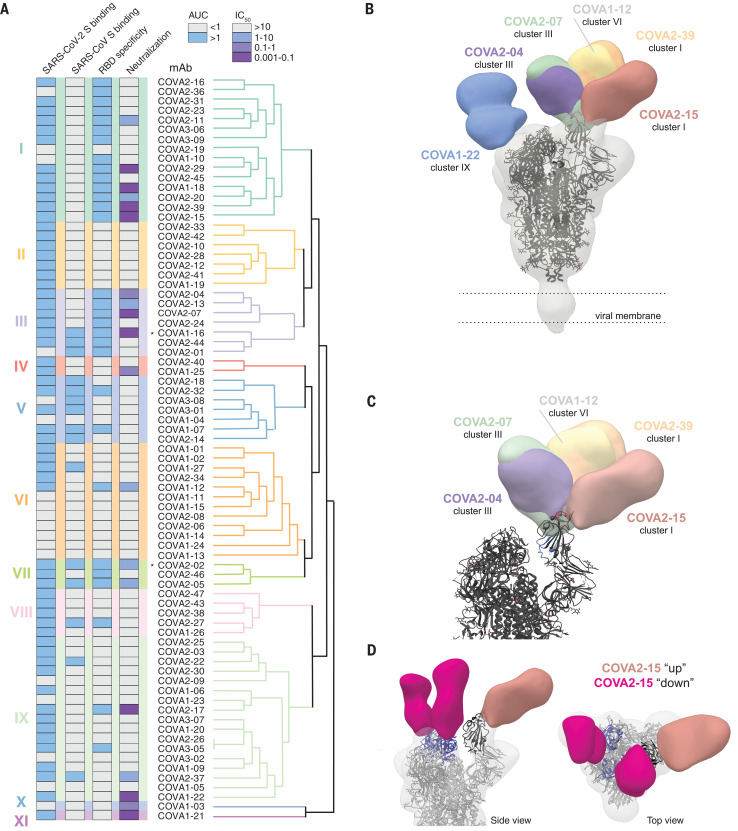
Antigenic clustering of SARS-CoV-2 S protein–specific mAbs. (**A**) Dendrogram showing hierarchical clustering of the SPR-based cross-competition heat map (table S2). Clusters are numbered I to XI and are depicted with color shading. ELISA binding to SARS-CoV-2 S protein, SARS-CoV S protein, and SARS-CoV-2 RBD as presented by AUC and neutralization IC_50_ (μg/ml) of SARS-CoV-2 is shown in the columns on the left. ELISA AUCs are shown in gray (AUC < 1) or blue (AUC > 1), and neutralization IC_50_ is shown in gray (>10 μg/ml), blue (1 to 10 μg/ml), violet (0.1 to 1 μg/ml), or purple (0.001 to 0.1 μg/ml). Asterisks indicate antibodies that cross-neutralize SARS-CoV pseudovirus. (**B**) Composite figure demonstrating binding of NTD-mAb COVA1-22 (blue) and RBD mAbs COVA2-07 (green), COVA2-39 (orange), COVA1-12 (yellow), COVA2-15 (salmon), and COVA2-04 (purple) to SARS-CoV-2 spike (gray). The spike model (PDB 6VYB) is fit into the density. (**C**) Magnification of SARS-CoV-2 spike comparing epitopes of RBD mAbs with the ACE2-binding site (red) and the epitope of mAb CR3022 (blue). (**D**) Side (left) and top (right) views of the 3D reconstruction of COVA2-15 bound to SARS-CoV-2 S protein. COVA2-15 binds to both the down (magenta) and up (salmon) conformations of the RBD. The RBDs are colored blue in the down conformation and black in the up conformation. The angle of approach for COVA2-15 enables this broader recognition of the RBD while also partially overlapping with the ACE2-binding site and therefore blocking receptor engagement.

To identify and characterize the antigenic sites on the S protein and their interrelationships, we performed SPR-based cross-competition assays using S protein, followed by clustering analysis. We note that competition clusters do not necessarily equal epitope clusters but the analysis can provide clues as to the relationship between mAb epitopes. We identified 11 competition clusters, of which nine contained more than one mAb and two contained only one mAb (clusters X and XI; Fig. 5A and fig. S5). All nine multiple-mAb clusters included mAbs from at least two of the three patients, emphasizing that these clusters represent common epitopes targeted by the human humoral immune response during SARS-CoV-2 infection. Three clusters included predominantly RBD-binding mAbs (clusters I, III, and VII), with cluster I forming two subclusters. These three clusters were confirmed by performing cross-competition experiments with soluble RBD instead of complete S protein (fig. S5B). Four clusters (V, VI, XIII, and IX) included predominantly mAbs that did not interact with RBD, and clusters II, IV, X, and XI consisted exclusively of non-RBD mAbs. mAbs with diverse phenotypes (e.g., RBD and non-RBD–binding mAbs) merged together in multiple clusters, suggesting that these mAbs might target epitopes bridging the RBD and non-RBD sites or that they sterically interfere with each other’s binding as opposed to binding to overlapping epitopes. Although clusters II, V, and VIII contained only mAbs incapable of neutralizing SARS-CoV-2, clusters I, III, IV, VI, and VII included both non-NAbs and NAbs. Cluster V was formed mostly by non-RBD–targeting mAbs that also bound to SARS-CoV. However, these mAbs were not able to neutralize either SARS-CoV-2 or SARS-CoV, suggesting that these mAbs target a conserved non-neutralizing epitope on the S protein. Finally, the two non-RBD mAbs COVA1-03 and COVA1-21 formed single-mAb competition clusters (clusters X and XI, respectively) and showed an unusual competition pattern, because binding of either mAb blocked binding by most of the other mAbs, but not vice versa (figs. S5 and S6 and table S2). We hypothesize that these two mAbs allosterically interfere with mAb binding by causing conformational changes in the S protein that shield or impair most other mAb epitopes. COVA1-21 also efficiently blocked virus infection without blocking ACE2, suggesting an alternative mechanism of neutralization than blocking ACE2 engagement (fig. S4C). The SPR-based clustering was corroborated using biolayer interferometry competition assays on a subset of NAbs (fig. S6). Overall, our data are consistent with the previous identification of multiple antigenic RBD sites for SARS-CoV-2 and additional non-RBD sites on the S protein, as described for SARS-CoV and MERS-CoV (*32*, *42*).

To visualize how selected NAbs bound to their respective epitopes, we generated Fab–SARS-CoV-2 S complexes that were imaged by single-particle negative-stain electron microscopy (EM; Fig. 5, B and C, and fig. S7). We obtained low-resolution reconstruction with six Fabs, including five RBD-binding Fabs from three different competition clusters. COVA1-12 overlapped highly with the epitope of COVA2-39, whereas COVA2-04 approached the RBD at a different angle somewhat similar to that of the cross-binding SARS-CoV–specific mAb CR3022 (*42*). The EM reconstructions confirmed the RBD as the target of these NAbs but revealed a diversity in approach angles (Fig. 5B). Furthermore, whereas four RBD NAbs interacted with a stoichiometry of one Fab per trimer, consistent with one RBD being exposed in the “up state” and two in the less accessible “down state” (*13*, *43*), COVA2-15 bound with a stoichiometry of three per trimer (fig. S7). COVA2-15 was able to bind RBD domains in both the up and down state (Fig. 5D). In either conformation, the COVA2-15 epitope partially overlapped with the ACE2-binding site, and therefore the mAb blocks receptor engagement. The higher stoichiometry of this mAb may explain its unusually strong neutralization potency. None of the epitopes of the five RBD Fabs overlapped with that of CR3022, which is unable to neutralize SARS-CoV-2 (*42*), although COVA2-04 does approach the RBD from a similar angle as CR3022. The sixth Fab for which we generated a three-dimensional (3D) reconstruction was from the non-RBD mAb COVA1-22 placed in competition cluster IX. EM demonstrated that this mAb bound to the NTD of S1. Such NTD NAbs have also been found for MERS-CoV (*44*).

## Conclusions

Convalescent COVID-19 patients showed strong anti–SARS-CoV-2 S protein–specific B cell responses and developed memory and antibody-producing B cells that may have participated in the control of infection and the establishment of humoral immunity. We isolated 19 NAbs that targeted a diverse range of antigenic sites on the S protein, of which two showed picomolar neutralizing activities (IC_50_ = 0.007 and 0.009 μg/ml or 47 and 60 pM, respectively) against authentic SARS-CoV-2 virus. This illustrates that SARS-CoV-2 infection elicits high-affinity and cross-reactive mAbs targeting the RBD as well as other sites on the S protein. Several of the potent NAbs had VH segments virtually identical to their germline origin, which holds promise for the induction of similar NAbs by vaccination because extensive affinity maturation does not appear to be a requirement for potent neutralization. The most potent NAbs both targeted the RBD on the S protein and fell within the same competition cluster, but were isolated from two different individuals and bore little resemblance genotypically. Although direct comparisons are difficult, the neutralization potency of these and several other mAbs exceeds the potencies of the most advanced HIV-1 and Ebola mAbs under clinical evaluation, as well as the approved anti-RSV mAb palivizumab (*45*). Through large-scale SPR-based competition assays, we defined NAbs that targeted multiple sites of vulnerability on the RBD and the additional previously undefined non-RBD epitopes on SARS-CoV-2. This is consistent with the identification of multiple antigenic RBD sites for SARS-CoV-2 and the presence of additional non-RBD sites on the S protein of SARS-CoV and MERS-CoV (*32*). Subsequent structural characterization of these potent NAbs will guide vaccine design, and simultaneous targeting of multiple non-RBD and RBD epitopes with mAb cocktails paves the way for safe and effective COVID-19 prevention and treatment.

## Supplementary Materials

science.sciencemag.org/content/369/6504/643/suppl/DC1 Materials and Methods Figs. S1 to S5 References (*46*–*56*) Tables S1 and S2 MDAR Reproducibility Checklist

## Appendix

View/request a protocol for this paper from *Bio-protocol*.
